# Predictive modeling of molecular interaction energies using topological and spectral entropies of zeolite AWW

**DOI:** 10.3389/fchem.2025.1543588

**Published:** 2025-03-18

**Authors:** Pancras Peter, Joseph Clement, Micheal Arockiaraj, Kavin Jacob

**Affiliations:** ^1^ Department of Mathematics, School of Advanced Sciences, Vellore Institute of Technology, Vellore, India; ^2^ Department of Mathematics, Loyola College, Chennai, India

**Keywords:** degree indices, information entropy, eigenvalue, spectral entropy, HOMO–LUMO gap, global reactivity descriptors, AWW zeolite

## Abstract

Zeolites are extremely massive mineral crystals with complex frameworks, composed of internal porous structures with channels and cages. Open-framework aluminophosphates (AlPOs) are a significant class of inorganic crystalline compounds known for their diverse properties. Our study focuses on the topological aspects of zeolite frameworks using graph theoretical techniques, providing insights into computational chemistry. In this article, we compute various degree-based topological indices, information entropy, and spectral entropies of zeolite AWW using the bond partitioning approach to assess the complexity of the framework. Additionally, we present the HOMO–LUMO gap measures to evaluate the global chemical descriptors using the eigenvalues of the adjacency matrix of zeolite structures. Furthermore, we developed exponential and polynomial regression models using the obtained information entropy and spectral values to predict various potential energies of the framework. Based on the outcomes of the study, we infer that the information entropies and spectral value have a significant relationship with the potential energies.

## 1 Introduction

Zeolites are minerals with crystalline structures that have regular frameworks constructed by channels and pores an the molecular level. Zeolite catalysts are employed throughout gasoline production and in the fields of adsorption, ion exchange, heterogeneous catalysis, sensors, and medicine. These crystals are also frequently used in solar energy conversion (Mihaela and Ildiko, 2012). Zeolite frameworks have Si tetrahedral nodes (T-atoms) and T-O-T, a bond referring to the arrangement where T stands for a tetrahedrally coordinated atom, usually silicon (Si) or aluminum (Al), and O stands for oxygen. The oxygen atom acts as a bridge between two tetrahedrally coordinated sites. Replacing Si with Al or other tetrahedral atoms significantly alters the characteristics of zeolites. This changes the framework’s affinity to other cations, allowing for customization of ion-exchange characteristics and adsorption surfaces. The tetrahedral atoms’ three-dimensional geometry forms rings, cages, channels, and pores, resulting in different frameworks (Kapko et al., 2010; Liebau, 1983; Barrer, 1979). Because there were many distinct materials, some with a common framework but differing by the chemical proposition, we needed to categorize all the different crystalline aluminosilicate materials. As a result, the concept of structural variation came into existence (Liebau, 1983; Barrer, 1979).

**FIGURE 1:**
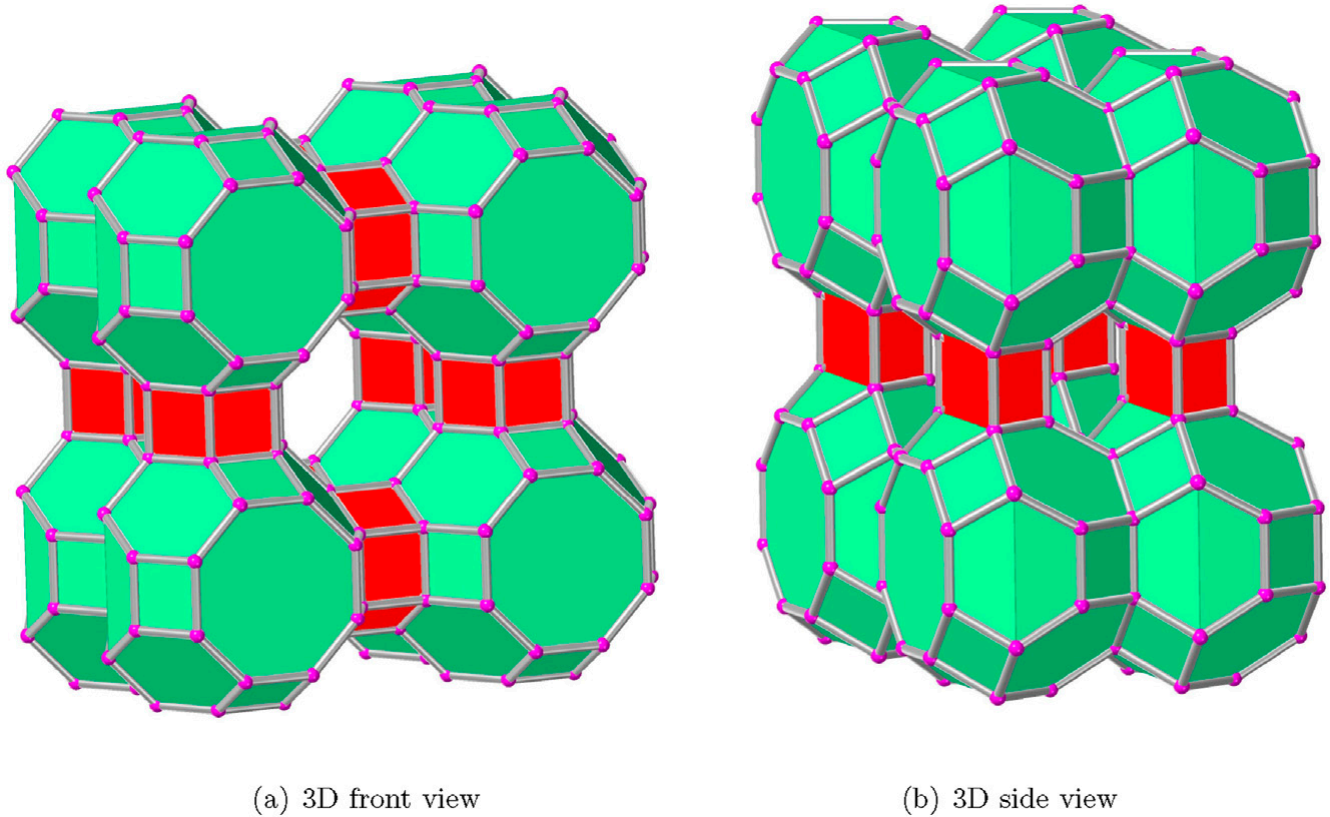
Polyhedral views of the zeolite AWW(2,2,2) framework.

The aluminophosphate crystalline sieves, denoted as ${\text{AlPO}}_{4}\;-n$ (where n corresponds to distinct structure type), marks the beginning of the era of open-framework aluminophosphates as a significant group of zeolites together with associated microporous materials (Wilson et al., 1982; Richardson et al., 1989). ${\text{AlPO}}_{4}\;-n$ is constructed based on the precise alternation of ${\text{AlO}}_{4}$ and ${\text{PO}}_{4}$ tetrahedra through corner exchange to generate a framework that is neutral and has an Al/P ratio of 1. Microporous aluminophosphates were discovered (Yu and Xu, 2006), and the extensive range of framework compositions and potential uses in catalysis, adsorption, and assembly have attracted much curiosity. The rational synthesis of novel materials has become increasingly significant in the field of materials chemistry (Richardson et al., 1989). Zeolite ${\text{AlPO}}_{4}\;-22$ or AWW has an innovative topological structure, which is depicted in Figure 1; it has an equal number of two new polyhedral units that share faces. In this study, the AWW zeolite framework is represented by a PDB file containing Si atoms in tetrahedral coordination linked by O atoms. To model Al and P atoms, specific Si atoms were substituted based on chemical rules, ensuring charge balance and structural consistency. The modified framework, comprising Al, P, and the remaining Si atoms, was used for topological characterization within the AWW topology. Topological measures such as degree and degree sum primarily focus on the contributions of heavier atoms like Al and P, while lighter O atoms act as linkers, indirectly influencing the connectivity. This distinction is significant because the structural and functional properties of zeolite materials largely depend on the ratio of heavy atoms.

A chemical composition graph is used to demonstrate the basic structure of a chemical compound (Balaban, 1985; Sato, 1991; Chandler, 2019). The vertices represent each of the atoms of the compound, and the edges indicate the bonds that occur among them. A topological index is used to measure the relationship between a chemical compound’s structure and various physical, chemical, or biological characteristics. Degree-based descriptors are extensively used topological descriptors with applications in computational chemistry, whereas topological indices based on neighborhood degree sum are capable of accurately determining most physicochemical parameters (Gutman and Tošović, 2013; Mondal et al., 2019; Ramane et al., 2021; Ullah et al., 2024; Mondal et al., 2021).

Topological descriptors for zeolites were developed to enhance machine learning, optimization, and algorithmic techniques (Krivovichev, 2013; Arockiaraj et al., 2022a; Arockiaraj et al., 2022b; Arockiaraj et al., 2021; Jacob et al., 2023). To better understand the basis for relating the structure and property to the synthesis technique, experimental and computational methods have been closely linked in modern zeolite synthesis. The zeolite framework influences its physicochemical properties such as adsorption, phase transformation, complexity, and chirality of molecules. Recent studies indicate that the topological index quantifies the relationship between zeolite molecular structures and a wide range of physicochemical characteristics (Jacob et al., 2023; Peter and Clement, 2023; Jacob and Clement, 2024; Peter and Clement, 2024; Prabhu et al., 2020).

The complexity of networks can be evaluated using statistical information measures (Bonchev, 1983; Anand and Bianconi, 2009; Mowshowitz, 1968a). Shannon’s information theory was used to develop the universal quantitative measurements of structural and chemical complexity, which are helpful for the investigation of several mineralogical, crystallographic, and chemical processes (Jacob et al., 2023; Dehmer and Mowshowitz, 2011; Krivovichev, 2012; Shannon, 1948). The probability of electrons in molecules and the chemical bonds between molecules have been effectively studied using information theory concepts (Nalewajski, 2006; Nalewajski, 2014). The communication theory for the chemical connection employs the basic information entropy of molecular systems in the atoms-in molecules, orbital, or local levels to ascertain electron probability distributions (Nalewajski, 2006; Nalewajski, 2014; Nalewajski, 2011). The information entropy (IE) method is used to quantitatively evaluate the complexity of zeolite structures and extract structural data from networks with numerous vertices (Krivovichev, 2013). The concept of structural information content based on the partitioning of vertex orbits was originally used to measure the complexity as well as the characteristics of the structure of molecular graphs (Rashevsky, 1960; Mowshowitz, 1968b; Dehmer, 2008).

The model obtained good accuracy and provided information about the connection between the zeolite framework structure and their mechanical stability (Evans and Coudert, 2017). The connections between zeolite frameworks and their characteristics are complicated, making an in-depth experimental investigation for novel zeolites necessary (Burtch et al., 2014). Various density functional theory computational techniques for determining zeolite characteristics have been established (Ranjan et al., 2023; Román-Román and Zicovich-Wilson, 2015; Fischer and Angel, 2017; Balasubramanian, 2023a; Balasubramanian, 2023b). Determining the zeolite lattice energy linked to formation enthalpy can help understand zeolite stability and structure, although computations are expensive but accurate (Román-Román and Zicovich-Wilson, 2015; Fischer and Angel, 2017; Stacey et al., 2023). Machine learning techniques can help determine the characteristics of zeolites by learning from a vast collection of known zeolites and their characteristics. It was demonstrated that some structural descriptors are suitable to characterize the lattice energy of zeolites in a comprehensible manner using a linearized equation, focusing on the intricate connection between structural features (Jacob et al., 2023; Peter and Clement, 2023; Jacob and Clement, 2024; Balestra et al., 2024). Electronic structural calculations help us understand how zeolite structures influence their performance. Long-range and short-range interaction energies significantly affect the geometry of the zeolite framework and their stability of transition states and adsorption behavior. Notably, long-range zeolite electrostatic interactions play a crucial role in describing transition-state structures and in predicting experimentally determined activation energies (Ranjan et al., 2023; Román-Román and Zicovich-Wilson, 2015; Fischer and Angel, 2017; Mansoor et al., 2018).

This article focuses on developing generalized expressions for descriptors such as degree-based and degree-sum-based measures specifically for the AWW zeolite structure, with any arbitrary dimension used to obtain structural data. A detailed analysis of bond information and spectral entropies is presented to evaluate the complexity of the AWW zeolite. Furthermore, we incorporated the exponential regression models that establish relationships between the measured entropies and molecular interaction energies, offering deeper insights into the energy and structural characteristics of zeolite. Global chemical reactivity descriptors and the spectral properties are also calculated using the eigen values of AWW zeolite structures.

## 2 Computational methods of the molecular topological index

Let $G=\left(V\left(G\right),E\left(G\right)\right)$ be a simple undirected graph. The number of atoms and bonds between atoms in the graph G is denoted by |V(G)| and |E(G)|, respectively. The degree of an atom u∈V(G) is given by $d_{u}$ , the number of bonds that are adjacent to atoms u and $ds_{u}$ can be described by the sum of the degrees of the neighborhood atoms of u, and it can be expressed as $ds_{u}=\sum_{v\in N_{G}\left(u\right)}d_{v}$ , where $N_{G}\left(u\right)=\left\{v\in V\left(G\right):uv\in E\left(G\right)\right\}$ .

For ψ∈{d,ds} , we define the degree and degree sum-type molecular topological indices (Gutman and Tošović, 2013; Mondal et al., 2019; Ramane et al., 2021; Gutman, 2013; Leszczynski, 2012) with the following expression:

$$T^{\psi }\left(G\right)=\underset{uv\in E\left(G\right)}{\sum }T^{\psi }\left(uv\right),$$ (1)

where $T^{\psi }\left(uv\right)$ is a structural function of the molecular topological index with respect to the edge of molecular graph, for example, $T^{\psi }\left(uv\right)$ defined for the first Zagreb $M_{1}^{\psi }\left(uv\right)=\psi_{u}+\psi_{v}$ , second Zagreb $M_{2}^{\psi }\left(uv\right)=\psi_{u}\psi_{v}$ , Randić $R^{\psi }\left(uv\right)=\frac{1}{\sqrt{\psi_{v}\psi_{u}}}$ , atom bond connectivity $ABC^{\psi }\left(uv\right)$ = $\sqrt{\frac{\psi_{u}+\psi_{v}-2}{\psi_{u}\psi_{v}}}$ , harmonic $H^{\psi }\left(uv\right)$ = $\frac{2}{\psi_{u}+\psi_{v}}$ , sum-connectivity $SC^{\psi }\left(uv\right)$ = $\frac{1}{\sqrt{\psi_{u}+\psi_{v}}}$ , hyper-Zagreb $HM^{\psi }\left(uv\right)$ = ${\left(\psi_{u}+\psi_{v}\right)}^{2}$ , geometric–arithmetic $GA^{\psi }\left(uv\right)$ = $2\frac{\sqrt{\psi_{u}\psi_{v}}}{\psi_{u}+\psi_{v}}$ , irregularity ${\text{irr}}^{\psi }\;\left(uv\right)$ = $\left|\psi_{u}-\psi_{v}\right|$ , sigma $\sigma^{\psi }\left(uv\right)$ = ${\left(\psi_{u}-\psi_{v}\right)}^{2}$ , forgotten $F^{\psi }\left(uv\right)$ = ${\left(\psi_{u}+\psi_{v}\right)}^{2}-2\left(\psi_{u}\psi_{v}\right)$ , augmented Zagreb $AZ^{\psi }\left(uv\right)\;{\left(\frac{\psi_{u}\psi_{v}}{\psi_{u}+\psi_{v}-2}\right)}^{3}$ , symmetric division degree $SDD^{\psi }\left(uv\right)$ = $\frac{{\left(\psi_{u}+\psi_{v}\right)}^{2}-2\left(\psi_{u}\psi_{v}\right)}{\psi_{u}\psi_{v}}$ , and Sombor $SO^{\psi }\left(uv\right)=\sqrt{\psi_{u}^{2}+\psi_{v}^{2}}$ .

The molecular topological descriptors based on the degree and degree sum are obtained via the atomic valences of the framework’s chemical bonds. Various degree-type topological indices have been developed and employed extensively in molecular modeling of a wide range of zeolite framework characteristics and activities (Arockiaraj et al., 2022a; Jacob et al., 2023; Zhang et al., 2024; Peter and Clement, 2023). Self-powered multiplicative topological indices of chemical structure are derived from the product of edge functions associated with the degrees of end vertices of an edge (Kavitha et al., 2021). These indices have been developed using exponential vertex degree-based descriptors (Rada, 2019). Therefore, one can develop a self-powered multiplicative version of degree and degree sum-based topological indices using the following general form:

$${T^{\psi }}^{\hat{s}*}\left(G\right)=\underset{uv\in E\left(G\right)}{\prod }{\left[T^{\psi }\left(uv\right)\right]}^{\left[T^{\psi }\left(uv\right)\right]}.$$ (2)

The edge partition technique is applied to derive the various degree and degree sum-based molecular topological indices. The sizes of the partitions on the basis of end vertex degrees are denoted by d(p,q), and the sizes of the partitions on the basis of end vertex degree sum are denoted by ds(p,q) . These quantities are explicitly presented as follows:

• $d\left(p,q\right)=\left|\left\{uv\in E\left(G\right):\left(d_{u},d_{v}\right)=\left(p,q\right),\;\forall p,q\geq 1\right\}\right|$

• $ds\left(p,q\right)=\left|\left\{uv\in E\left(G\right):\left(ds_{u},ds_{v}\right)=\left(p,q\right),\;\forall p,q\geq 1\right\}\right|$ .

By considering $\Delta =\max_{u\in V\left(G\right)}\psi_{u}$ and $C=\left\{\left(p,q\right)\in N\times N:1\leq p\leq q\leq \Delta^{2}\right\}$ , the generalized form of degree and neighborhood degree sum molecular topological indices Equations 1, 2 can be modified as Equations 3–6.

$$T^{d}\left(G\right)=\underset{\left(p,q\right)\in C}{\sum }d\left(p,q\right)\left[T^{d}\left(pq\right)\right],$$ (3)

$$T^{ds}\left(G\right)=\underset{\left(p,q\right)\in C}{\sum }ds\left(p,q\right)\left[T^{ds}\left(pq\right)\right],$$ (4)

$${T^{d}}^{\hat{s}*}\left(G\right)=\underset{\left(p,q\right)\in C}{\prod }{\left[{\left(T^{d}\left(pq\right)\right)}^{\left(T^{d}\left(pq\right)\right)}\right]}^{d\left(p,q\right)},$$ (5)

$${T^{ds}}^{\hat{s}*}\left(G\right)=\underset{\left(p,q\right)\in C}{\prod }{\left[{\left(T^{ds}\left(pq\right)\right)}^{\left(T^{ds}\left(pq\right)\right)}\right]}^{ds\left(p,q\right)}.$$ (6)

### 2.1 Bond information entropy

The studies (Krivovichev, 2013; Krivovichev, 2012; Kaußler and Kieslich, 2021; Krivovichev, 2016) used the following equation to determine the quantity of structural Shannon’s information of the crystal structure:

$$IE\left(G\right)=-\overset{n}{\underset{i=1}{\sum }}\rho_{i}\log\rho_{i},$$ (7)

where i represents the number of different crystallographic orbits in the structure and $p_{i}$ is the random choice probability for an atom from the $i^{th}$ crystallographic orbit, in other terms $\rho_{i}=\frac{m_{i}}{n}$ , where $m_{i}$ and n are the multiplicity of the crystallographic orbit and the number of atoms in the primitive unit cell, respectively.

Now, Equation 7 will be modified to characterize the structural characteristics of the zeolite framework. The bonds of the zeolite framework are taken into account, and each of them receives a probability value based on the topological indices. The topological information entropy measures for degree type are defined using that molecular topological index T (Bonchev, 1983).

$$IE_{T}\left(G\right)=-\underset{e\in E\left(G\right)}{\sum }\rho_{r}\left(e\right)\log\rho_{r}\left(e\right),$$

where $\rho_{r}\left(e\right)$ is the probability function of an edge that is based on the degree which is given by

$$\rho_{r}\left(e\right)=\frac{T^{d}\left(uv\right)}{\sum_{uv\in E\left(G\right)}T^{d}\left(uv\right)}=\frac{T^{d}\left(uv\right)}{T^{d}\left(G\right)}.$$

The information entropy measure based on the degree sum molecular topological descriptors is obtained by replacing $T^{d}$ with $T^{ds}$ . As observed in (Kazemi, 2016), the study introduces a new method for calculating graph entropy, utilizing degree-based topological indices as edge weights to evaluate the complexity and uncertainty of a graph’s structure. We could develop and manufacture an extensive range of novel materials with the desired characteristics using the information entropy concept, which was initially applied to inorganic and organic materials (Jacob et al., 2023; Peter and Clement, 2023; Abraham et al., 2022; Rahul et al., 2022; Kalaam et al., 2024), and study the compositional intricacy and structural disorder to change the structural and functional characteristics. Therefore, by using different topological indices, we obtain various entropy values given as follows:

$$IE_{T^{\psi }}\left(G\right)=\log\left(T^{\psi }\left(G\right)\right)-\frac{1}{T^{\psi }\left(G\right)}\underset{uv\in E\left(G\right)}{\sum }\left[T^{\psi }\left(uv\right)\log\left(T^{\psi }\left(uv\right)\right)\right].$$ (8)

By reformulating Equation 8 using self-powered multiplicative degree-based indices, as described in (Kavitha et al., 2021), we could establish a relationship between the self-powered multiplicative indices and the entropy computation typically associated with bond additive indices.

$$IE_{T^{\psi }}\left(G\right)=\log\left(T^{\psi }\left(G\right)\right)-\frac{1}{T^{\psi }\left(G\right)}\log\left[{T^{\psi }}^{\hat{s}*}\left(G\right)\right].$$ (9)

The developed Equations 8, 9 are used to measure the various bond information entropy values of AWW zeolite. Furthermore, the measured information entropy was used to develop the possibility of machine learning of the AWW zeolite structure for studying the properties and activities (Arockiaraj et al., 2022a; Peter and Clement, 2023; Jacob and Clement, 2024; Peter and Clement, 2024).

### 2.2 Spectral information entropies

To define the spectral entropies for each edge uv∈E(G) , we first construct a symmetric square matrix of order |V(G)| using the degree-based topological index of the graph as defined below (Bozkurt et al., 2010; Ghanbari, 2022; Rodríguez and Sigarreta, 2016):

$$M\left(T^{d}\right)=\left\{\begin{array}{cc} 0\; & if\;the\;vertices\;u\;and\;v\;of\;G\;are\;not\;adjacent\\ T^{d}\left(uv\right)\; & if\;the\;vertices\;u\;and\;v\;of\;G\;are\;adjacent \end{array}\right.,$$ (10)

where $T^{d}\left(uv\right)$ is a structural function of topological indices with respect to an edge, as defined in Section 2. For example, the matrix with respect to the sum connectivity index is defined as follows:

$$M\left(SC^{d}\right)=\left\{\begin{array}{cc} 0\; & if\;the\;vertices\;u\;and\;v\;of\;G\;are\;not\;adjacent\\ \frac{1}{\sqrt{d_{u}+d_{v}}}\; & if\;the\;vertices\;u\;and\;v\;of\;G\;are\;adjacent \end{array}\right..$$

The study of graph energy obtained from the topological matrix indices offers insights into the structural properties of graphs, with potential applications in chemistry and communication networks (Bozkurt et al., 2010; Ghanbari, 2022; Rodríguez and Sigarreta, 2016; Chen et al., 2014; Hayat et al., 2024; Hui et al., 2022; Kosar et al., 2023). The novelty of this section is that we use the computed eigen values of the topological matrix indices of the zeolite framework to measure the spectral information entropies. Let $\lambda_{1}^{d},\lambda_{2}^{d},\lambda_{3}^{d},\ldots ,\lambda_{r}^{d}$ be non-zero eigenvalues of the degree-based molecular topological index matrices obtained from Equation 10. Then, in order to compute the spectral entropies, we now modify Equation 7 as follows:

$$IE_{M\left(T^{\lambda^{d}}\right)}\left(G\right)=-\overset{r}{\underset{i=1}{\sum }}\frac{\left|\lambda_{i}^{d}\right|}{\sum_{j=1}^{r}|\lambda_{j}^{d}|}\log\left(\frac{\left|\lambda_{i}^{d}\right|}{\sum_{j=1}^{r}|\lambda_{j}^{d}|}\right),$$ (11)

where the ratio $\frac{\left|\lambda_{i}^{d}\right|}{\sum_{j=1}^{r}|\lambda_{j}^{d}|}$ is the random choice probability of eigen values. The degree sum-based entropies of the zeolite AWW can be measured using Equation 11 by exchanging $T^{\lambda^{ds}}$ for $T^{\lambda^{d}}$ .

## 3 Molecular topological indices of zeolite AWW

**FIGURE 2:**
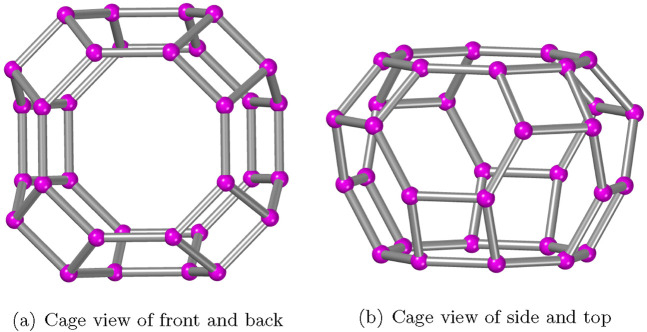
Primary cages in the AWW zeolite material.

**FIGURE 3:**
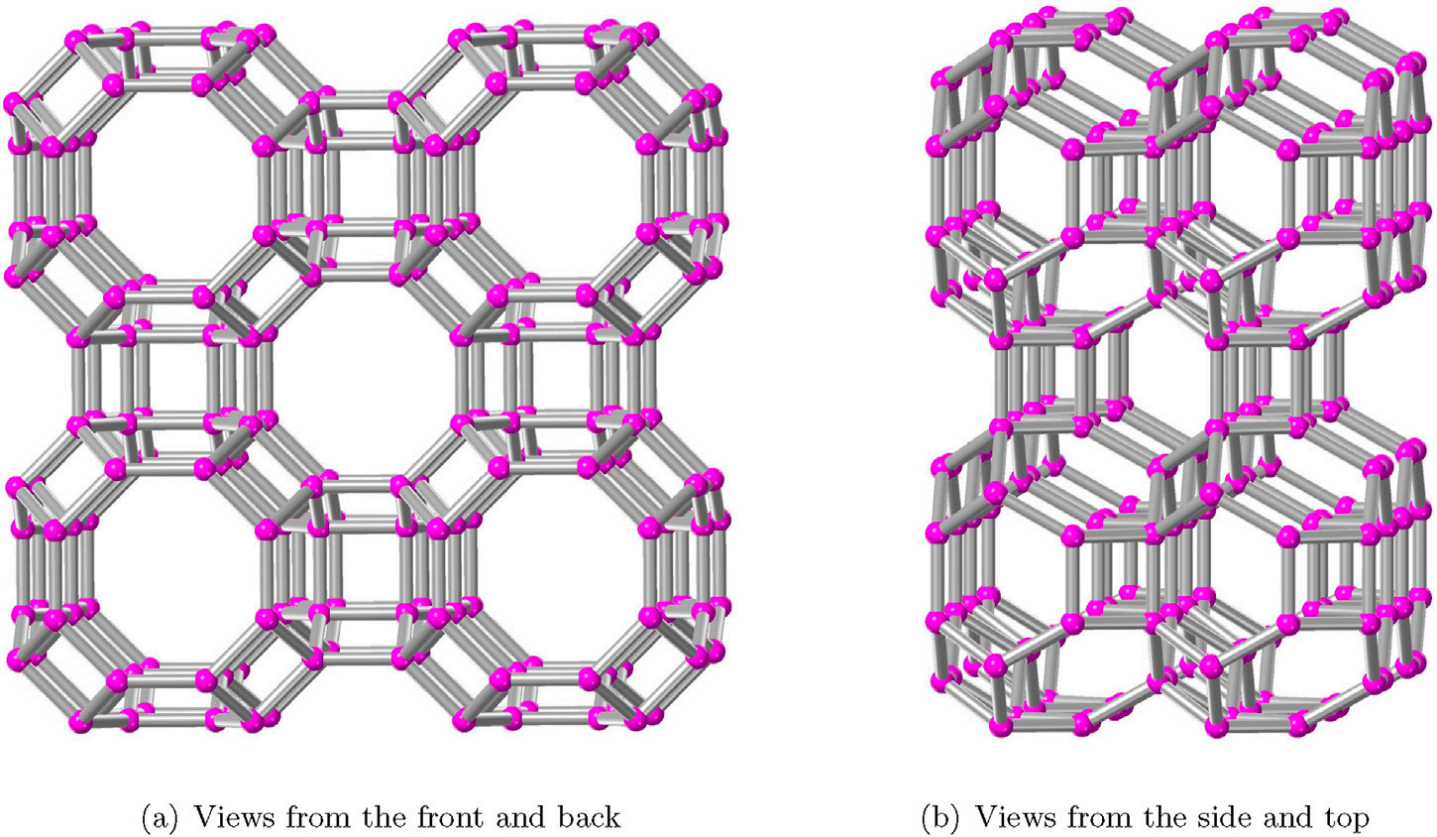
AWW(2,2,2) framework.

The AWW zeolite structures are characterized by using the measurements provided in Section 2. As depicted in Figure 2, the cavities serve as the primary building component for the zeolite AWW molecular communication system, which has 32 vertices and 48 edges connected by eight squares and two octagons, such that some bonds are shared. In order to form a three-dimensional molecular structure, the cavities of AWW are arranged in the j×k×l configuration. This leads to the crystal lattice, which is represented by AWW(j,k,l), as shown in Figure 3, with j,k , and l indicating the number of cavities arranged along the bottom to top, along left to right, and front to back copies, accordingly. Since translational symmetry repeats a single unit cell an infinite number of times to fill the pores and topological indices are numerical values that cannot be generalized in terms of infinity, we have considered point symmetry and not translational symmetry.

Figure 3 depicts the polyhedral structural growth of AWW. Figure 2 shows the primary cavities that are interconnected to generate the AWW(2,2,2) zeolite. Two types of polyhedral structures are considered in Figure 3: the front and back view frameworks. These are generated by connecting the two cavities via a tunnel and sharing all six rings between them. In contrast, the top- and side-view frameworks are directly connected by all eight rings and connected by the tunnels between the cavities. The total number of vertices (T-atoms) and edges (T-O-T bonds) in zeolite AWW(j,k,l) are 8jk(3l+1) and 4[12jkl+2jk−jl−kl] for j,k,l≥1 .

### 3.1 Degree-type molecular descriptors of *AWW* zeolite

We employed the edge partition technique according to the degrees of the end atoms of bonds, and the degree sum of the end atoms of bonds used to generate different degree-based molecular topological descriptors of zeolite AWW(j,k,l) for j,k,l≥1 is listed below.

Bond degree partitions:

• d(3,3)=2[8jk+3(jl+kl)+4(j+k)+2l]

• d(3,4)=4[4jk+3(jl+kl)−4(j+k)−2l]

• d(4,4)=2[24jkl−12jk−11(jl+kl)+4(j+k)+2l]

• ds(9,9)=10[j+k+2]

Bond degree-sum partitions (when j,k,l≥2 ):

• ds(9,10)=4[3j+3k−2]

• ds(10,10)=2[8jk+jl+kl−5(j+k)+6l−10]

• ds(10,14)=4[jl+kl+j+k+2l−6]

• ds(10,15)=4[4jk−3(j+k)+2]

• ds(11,9)=4[j+k−2]

• ds(11,10)=4(j+k−2)(l−2)

• ds(11,14)=4(j+k−2)(l−1)

• ds(11,15)=4(j+k−2)(l−1)

• ds(14,14)=2[jl+kl+2l−4]

• ds(14,16)=4l[j+k−2]

• ds(15,15)=2[8jk+jl+kl−5(j+k)−2l+2]

• ds(15,16)=4[4jk+2(jl+kl)−7(j+k)−4l+10]

• ds(16,16)=2[24jkl−28jk−19(jl+kl)+23(j+k)+14l−18]

Now let us obtain the molecular topological indices using Equation 1, and the estimated bond degree and bond degree sum partition are obtained from the above-listed partitions. The results of the zeolite AWW(j,k,l) are shown in Theorems 3.1, 3.2.

Since the results from Theorem 3.2 are applicable for j,k,l≥2 , we present the neighborhood degree sum-based bond partitions of two-dimensional zeolite AWW, i.e., l=1 and j,k≥1 .

Bond degree-sum partitions (when l=1 and j,k≥1 ):

• ds(9,9)=10(j+k)+28

• ds(14,14)=2(j+k)−4

• ds(10,10)=4(j+k)+16(j−1)(k−1)−8

• ds(10,14)=8(j+k)−16

• ds(10,15)=4(j+k)+16(j−1)(k−1)−8

• ds(14,15)=4(j+k)−8

• ds(9,10)=16(j+k)−32

• ds(15,15)=4(j+k)+24(j−1)(k−1)−8

By using the same procedure and the above-listed partitions, we can calculate the neighborhood degree sum-based indices of two-dimensional zeolite AWW.

Theorem 3.1 *Let* G *denote the AWW* (j,k,l) *zeolite framework,* j,k,l≥1 *. Then, the degree-form molecular topological descriptors are*

1. $M_{1}^{d}\left(G\right)=384jkl+16jk-56jl-56kl$

2. $M_{2}^{d}\left(G\right)=768jkl-48jk-154\left(jl+kl\right)+8\left(j+k\right)+4l$

3. $R^{d}\left(G\right)=12jkl+3.95213548685034jk$ −0.0358983848622456(jl+kl)+0.0478645131496611 (j+k)+0.0239322565748306l

4. $ABC^{d}\left(G\right)=29.3938769133981jkl+6.29768379985404jk$ −1.72622689289264(jl+kl)−0.0956427709867551(j+k) −0.0478213854933776l

5. $H^{d}\left(G\right)=\frac{1}{420}\left[1050jkl+140jk-15\left(jl+kl\right)+26\left(j+k\right)+20l\right]$

6. $SC^{d}\left(G\right)=16.9705627484771jkl+4.09412284133087jk$ −0.793111174158119(jl+kl)+0.0469818803094588(j+k) +0.0234909401547294l

7. $HM^{d}\left(G\right)=3072jkl-176jk-604\left(jl+kl\right)+16\left(j+k\right)+8l$

8. $GA^{d}\left(G\right)=48jkl+7.83589309777259jk$ −4.12308017667056(jl+kl)+0.16410690222741(j+k) +0.0820534511137048l

9. $irr^{d}\left(G\right)=\sigma^{d}\left(G\right)=16jk+12\left(jl+kl\right)-16\left(j+k\right)-8l$

10. $F^{d}\left(G\right)=1536jkl-80jk-296\left(jl+kl\right)$

11. $SDD^{d}\left(G\right)=\frac{1}{6}\left[120jkl+12jk-9\left(jl+kl\right)-5\left(j+k\right)-4l\right]$

12. $AZ^{d}\left(G\right)=\frac{1}{54000}\left[10240000jkl-2368000jk\right.$ $\left.-2689024\left(jl+kl\right)+555008\left(j+k\right)+584407l\right]$

13. $SO^{d}\left(G\right)=271.529003975634jkl+12.1177490060914jk$ −38.9949493661167(jl+kl)−0.804040507106677j −0.804040507106677k−0.402020253553339l

Theorem 3.2 *Let* G *denote the AWW* (j,k,l) *zeolite framework,* j,k,l≥2 *. Then, the degree-sum-form molecular topological descriptors are*

1. $M_{1}^{ds}\left(G\right)=1536jkl-96jk-308\left(jl+kl\right)+16\left(j+k\right)+8l$

2. $M_{2}^{ds}\left(G\right)=12288jkl-2896jk-3594\left(jl+kl\right)+696\left(j+k\right)+308l-24$

3. $R^{ds}\left(G\right)=3jkl+1.50585675513967jk+0.238024998461628\left(jl+kl\right)$ +0.140745376492254(j+k)+0.0476253820709413l +0.0199696427492026

4. $ABC^{ds}\left(G\right)=16.431676725155jkl+5.08922741392934jk-0.211371474742995\left(jl+kl\right)$ +0.0995211198551319(j+k)+0.0356164974373255l +0.00466120021924266

5. $H^{ds}\left(G\right)=3jkl+1.4789247311828jk+0.225964197093229\left(jl+kl\right)$ +0.159886990014324(j+k)+0.0528335105754461l +0.0251943547868845

6. $SC^{ds}\left(G\right)=8.48528137423857jkl+2.67308563317728jk-0.0862161391140532\left(jl+kl\right)$ +0.131697210934123(j+k)+0.0427038837370124l +0.0178547409567882

7. $HM^{ds}\left(G\right)=49152jkl-11168jk-14184\left(jl+l\right)+2440\left(j+k\right)+1104l-64$

8. $GA^{ds}\left(G\right)=48jkl+7.66840752524941jk$ −4.15007191255512(jl+kl)+0.2499927179825(j+k) +0.0763565800425527l+0.0332182510198214

9. $irr^{ds}\left(G\right)=96jk+64\left(jl+kl\right)-88\left(j+k\right)-64l+32$

10. $\sigma^{ds}\left(G\right)=416jk+192\left(jl+kl\right)-344\left(j+k\right)-128l+32$

11. $F^{ds}\left(G\right)=24576jkl-5376jk-6996\left(jl+kl\right)+1048\left(j+k\right)+488l-16$

12. $SDD^{ds}\left(G\right)=96jkl+18.7333333333333jk$ −6.78008658008658(jl+kl)−2.05894660894661(j+k) −0.611255411255411l−0.266233766233766

13. $AZ^{ds}\left(G\right)=29826.1617777778jkl-10244.258617031jk$ −10484.839762917(jl+kl)+3201.8324609039(j+k) +1437.40740464836l−261.075052832933

14. $SO^{ds}\left(G\right)=1086.11601590254jkl-62.0984297085552jk$ −215.133170624545(jl+kl)+6.72793994101937(j+k) +4.0908228573993l−0.0601654666833156

For computing the degree and degree sum-type multiplicative self-powered molecular descriptors of the AWW zeolite, Equations 5, 6 were employed to generate the corresponding indices using the listed bond degree and bond degree sum partitions. For instance, the analytical expression for degree-based self-powered sum connectivity index is

$$\begin{array}{ll} {T^{d}}^{\hat{s}*}\left(G\right) & ={\left(T^{d}{\left(33\right)}^{T^{d}\left(33\right)}\right)}^{d\left(3,3\right)}\times {\left(T^{d}{\left(34\right)}^{T^{d}\left(34\right)}\right)}^{d\left(3,4\right)}\times {\left(T^{d}{\left(44\right)}^{T^{d}\left(44\right)}\right)}^{d\left(4,4\right)}\\ {T^{d}}^{\hat{s}*}\left(G\right) & ={\left(\frac{1558910783423325}{2251799813685248}\right)}^{2\left[8jk+3\left(jl+kl\right)+4\left(j+k\right)+2l\right]}\\ & \times {\left(\frac{779568926258033}{1125899906842624}\right)}^{4\left[4jk+3\left(jl+kl\right)-4\left(j+k\right)-2l\right]}\\ & \times {\left(\frac{390508353708945}{562949953421312}\right)}^{2\left[24jkl-12jk-11\left(jl+kl\right)+4\left(j+k\right)+2l\right]}. \end{array}$$

Furthermore, the information entropy measures multiplicative self-powered for bond additive degree, and bond additive degree sum topological indices are easily obtained using Equation 9.

## 4 Determining molecular interaction energies of AWW zeolite through entropy measures

### 4.1 Degree and degree sum-type information and spectral entropies

This section summarizes the information entropy of AWW zeolite and suggests possible development using the degree, degree sum-based information, and spectral entropies of Equations 8, 11 by assessing the mineral complexity given in Tables 1, 2. It can be very useful for selected dimensions for training in an attempt to analyze the information entropies for larger complex molecules. For all entropy measures, the $IE_{T^{d}}$ values are consistently higher than the $IE_{T^{ds}}$ values, indicating that $IE_{T^{ds}}$ incorporates more detailed structural aspects of the system, as shown in Table 1. Different entropy measures increase at different rates as j increases. Some information entropy measures are distributed unequally for each layer of AWW zeolite frameworks, except the geometric arithmetic degree and degree sum-based information entropy compared to others. Only the geometric arithmetic information entropy has a significant level of discrimination among all the measured information entropies. Geometric arithmetic entropy is more effective in discriminating the structural complexity because it integrates both local and global structural information, balances contributions across the framework, and is highly sensitive to connectivity variations. On the other hand, the discriminatory power is low for the second Zagreb, harmonic, and forgotten information entropies. This variation indicates that each measure might be sensitive to different aspects of structural complexity in the zeolite. In this study, we exclude the irr and sigma entropies as they are indeterminate with respect to bond additive degree and bond additive degree sum-type entropies.

**TABLE 1:** Information entropy measures of AWW(j,k,l) zeolite, when j=k=l .

Entropies		j=2	j=3	j=4	j=5	j=6	j=7	j=8
$IE_{M_{1}}$	d	5.9425	7.1603	8.0246	8.6949	9.2425	9.7055	10.1065
	ds	5.9273	7.1484	8.0151	8.6871	9.2359	9.6998	10.1014
$IE_{M_{2}}$	d	5.9188	7.1415	8.0096	8.6825	9.232	9.6964	10.0984
	ds	5.862	7.1004	7.978	8.657	9.2106	9.678	10.0823
$IE_{R}$	d	5.9425	7.1594	8.0234	8.6937	9.2414	9.7044	10.1055
	ds	5.927	7.1444	8.0102	8.6821	9.2311	9.6952	10.0972
$IE_{\mathrm{ABC}}$	d	5.9499	7.1664	8.0295	8.699	9.246	9.7085	10.1092
	ds	5.9457	7.1624	8.0261	8.6961	9.2435	9.7063	10.1071
${IE}_{\text{H}}$	d	5.9425	7.1594	8.0235	8.6938	9.2415	9.7045	10.1056
	ds	5.9268	7.1445	8.0104	8.6824	9.2314	9.6955	10.0975
$IE_{SC}$	d	5.9486	7.1652	8.0285	8.6982	9.2453	9.7079	10.1086
	ds	5.9446	7.1616	8.0255	8.6955	9.243	9.7059	10.1068
$IE_{HM}$	d	5.919	7.142	8.0101	8.683	9.2324	9.6967	10.0988
	ds	5.863	7.1018	7.9793	8.6582	9.2116	9.6789	10.0831
$IE_{GA}$	d	5.9506	7.167	8.0301	8.6995	9.2465	9.7089	10.1095
	ds	5.9506	7.167	8.0301	8.6995	9.2465	9.7089	10.1095
$IE_{F}$	d	5.9192	7.1424	8.0105	8.6834	9.2328	9.6971	10.0991
	ds	5.8638	7.103	7.9805	8.6592	9.2126	9.6797	10.0838
$IE_{\mathrm{SDD}}$	d	5.9505	7.1669	8.03	8.6994	9.2464	9.7089	10.1095
	ds	5.9503	7.1667	8.0298	8.6993	9.2463	9.7088	10.1094
$IE_{AZ}$	d	5.9246	7.1452	8.0121	8.6845	9.2336	9.6976	10.0995
	ds	5.7921	7.0514	7.9408	8.6271	9.1857	9.6566	10.0635
$IE_{SO}$	d	5.9425	7.1604	8.0247	8.695	9.2426	9.7056	10.1065
	ds	5.9274	7.1487	8.0154	8.6874	9.2362	9.7	10.1016

**TABLE 2:** Spectral entropy measures of AWW(j,k,l) zeolite, when j=k=l .

Entropies	j=2	j=3	j=4	j=5	j=6	j=7	j=8
$IE_{M\left(M_{1}^{\lambda^{d}}\right)}$	4.8234	5.9198	6.6881	7.3033	7.8158	8.244	8.626
$IE_{M\left(M_{2}^{\lambda^{d}}\right)}$	4.7986	5.8947	6.6636	7.2815	7.7965	8.2265	8.6103
$IE_{M\left(R^{\lambda^{d}}\right)}$	4.8259	5.9278	6.7003	7.3155	7.8276	8.2557	8.6368
$IE_{M\left(ABC^{\lambda^{d}}\right)}$	4.8318	5.9318	6.7024	7.3169	7.8285	8.2561	8.637
$IE_{M\left(H^{\lambda^{d}}\right)}$	4.8258	5.9281	6.7006	7.3158	7.8279	8.256	8.637
$IE_{M\left(SC^{\lambda^{d}}\right)}$	4.8308	5.9317	6.7028	7.3175	7.8291	8.2569	8.6377
$IE_{M\left(HM^{\lambda^{d}}\right)}$	4.798	5.8948	6.6639	7.2817	7.7968	8.2267	8.6105
$IE_{M\left(GA^{\lambda^{d}}\right)}$	4.8323	5.9317	6.7015	7.3159	7.8274	8.255	8.636
$IE_{M\left(F^{\lambda^{d}}\right)}$	4.7986	5.895	6.6641	7.2819	7.7971	8.2269	8.6107
$IE_{M\left(SDD^{\lambda^{d}}\right)}$	4.8319	5.9307	6.7006	7.315	7.8267	8.2542	8.6352
$IE_{M\left(AZ^{\lambda^{d}}\right)}$	4.8055	5.9012	6.6695	7.2866	7.8008	8.2306	8.6139
$IE_{M\left(SO^{\lambda^{d}}\right)}$	4.8233	5.9197	6.6881	7.3032	7.8158	8.244	8.6259

The characteristics of the AWW zeolite structure can be studied using the data provided in Table 1. This allows for the investigation of atomic chemical properties, structural complexity, diversity, similarity, modularity, chirality, and molecular structure information in crystal systems. In addition, in this study, degree spectral entropies were measured to assess the structural complexity of zeolite frameworks. The spectral degree-based entropies have the lowest discriminative values compared to degree and degree sum-based information entropies. This implies that spectral entropy focuses on specific modes or energy distributions within the structure rather than the overall disorder. The zero eigenvalue of the zeolite adjacency matrix is indeterminate in the spectral information entropy calculations from Equation 11 as it causes issues in logarithmic terms. To ensure accuracy, the zero eigenvalue must be excluded or addressed through normalization techniques.

Section 4.2 will address the prediction abilities of our measured bond information and spectral entropies obtained from the AWW zeolite structure.

### 4.2 Predictive model of various entropies with molecular interaction energies of AWW zeolite

Density functional theory (DFT) has limitations in calculating the molecular interaction energies for large AWW zeolite systems due to its high computational costs, difficulty in capturing long-range interactions, and challenges in modeling non-covalent interactions and strongly correlated electron systems. It also struggles with the flexibility of zeolite frameworks and dynamic effects (Cohen et al., 2012). These issues make DFT impractical for application in large zeolite systems, underscoring the need for predictive models like machine learning to efficiently estimate interaction energies, so there is another computational method to calculate the molecular energies of zeolites: force field method. Force-field methods, in contrast, offer an efficient, cost-effective alternative for studying zeolite structures. These methods allow for scalable simulations of large, complex frameworks and can provide insights into time-dependent behaviors such as adsorption, diffusion, and framework flexibility. Although they rely on parameterized potentials and may struggle with modeling larger zeolite structures, force-field methods, when tailored to specific systems, complement experimental data and fill gaps in case of limited experiments, thus playing a crucial role in advancing our understanding of zeolite properties (Dubbeldam et al., 2019; Jaramillo and Auerbach, 1999).

Determining the properties of zeolites experimentally is challenging due to their structural complexity, which includes intricate pore networks, large unit cells, and diverse atomic environments. These challenges are further compounded by the limited crystallinity, structural defects, and difficulties in synthesizing high-quality samples, all of which impede accurate measurements (Price et al., 2021). The unique crystal structure of the AWW zeolite, characterized by intricate cages and novel configurations, exemplifies this complexity and contributes to the limited availability of experimental data. Additionally, zeolite properties are highly sensitive to environmental factors such as temperature and pressure, complicating the reproducibility. Advanced techniques like X-ray diffraction, neutron diffraction, and nuclear magnetic resonance add to the difficulty, given their high costs and time requirements (Van Vreeswijk and Weckhuysen, 2022).

This subsection investigates the entropies of zeolite AWW using both the bond additive and bond additive sum indices. The results are then correlated with the physical properties of zeolites. For force-field calculations, the Molecular Mechanics 3 (MM3) and Universal Force Field (UFF) approaches are employed within the CRYSTAL computing tools. These tools are essential for analyzing the zeolite’s structural and energetic properties, enabling accurate calculation of molecular interaction energies such as $\left(E^{es}\right)$ , total energy $\left(E^{te}\right)$ , dispersion $\left(E^{\mathrm{dse}}\right)$ , dihedral $\left(E^{\mathrm{dhd}}\right)$ , two-body $\left(E^{2b}\right)$, and three-body $\left(E^{3b}\right)$ , which are associated with various AWW zeolite structures (Dovesi et al., 2005; Palmer, 2015). Consequently, the following Table 3 presents the experimental data calculated for AWW zeolite structures. With the goal of evaluating the measured information entropy’s predictive ability, data were obtained.

**TABLE 3:** Molecular interaction energy properties of AWW(j,k,l) , when j=k=l .

AWW(j,k,l)	|V|	|E|	Molecular interaction energies (eV) of AWW zeolites					
			$E^{es}$	$E^{\mathrm{dse}}$	$E^{te}$	$E^{\mathrm{dhd}}$	$E^{2b}$	$E^{3b}$
AWW (2,2,2)	224	384	0.7434	−4.7931	694.0131	22.5778	592.2638	83.2212
AWW (3,3,3)	720	1,296	6.1657	−17.6518	2372.0346	85.6196	1996.6792	301.2218
AWW (4,4,4)	1,664	3,072	13.7597	−43.6160	5652.7948	214.2766	4730.2483	738.1260
AWW (5,5,5)	3,200	6,000	20.2031	−87.2845	11070.4142	431.8839	9235.6949	1469.9167
AWW (6,6,6)	5,472	10,368	25.8579	−161.7722	20135.1759	804.6664	16768.2665	2698.1572
AWW (7,7,7)	8,624	16,464	36.4116	−246.1287	30472.7760	1227.2898	25333.1150	4122.0882
AWW (8,8,8)	12,800	24,576	46.1900	−370.5015	45532.4173	1851.7584	37810.5359	6194.4345

A detailed exponential fit analysis was performed between the properties provided in Table 3 and the information entropy values to determine the possible molecular energies and construct models for prediction. The AWW zeolite’s bond degree, degree sum, and spectral information entropy are taken into account for developing the following exponential fit prediction model:

$$P=x_{0}+y_{0}\times e^{\left(z_{0}\times IE_{T^{\psi }}\right)}.$$ (12)

where P represented that the potential energies, $x_{0}$ , $y_{0}$ , and $z_{0}$ are coefficients that significantly fit the exponential curve, and $IE_{T^{\psi }}$ information entropies of AWW are listed in Table 4. To test the prediction ability of spectral-based information entropy of the AWW zeolite structure, we substitute $IE_{M\left(T^{\lambda^{d}}\right)}$ with $IE_{T^{\psi }}$ in Equation 12. The exponential models were developed using Equation 12 to establish relationships between the data presented in Tables 1–3. The established exponential fit model is a good approximation for modeling the information entropy of the zeolite structure as a function of potential energies because the model attained the coefficient of determination $\left(R^{2}\right)$ as 0.99.

**TABLE 4:** Significant coefficients for exponential fit of entropies with molecular energies.

$IE_{T^{\psi }}$	P	$x_{0}$	$y_{0}$	$z_{0}$	$R^{2}$
$IE_{R^{d}}$	$E^{\mathrm{dhd}}$	−14.37505	0.08337	0.991	0.9995
$IE_{F^{ds}}$	$E^{\mathrm{dse}}$	2.44953	−0.01934	0.97843	0.9996
$IE_{R^{ds}}$	$E^{3b}$	−39.03459	0.30396	0.98325	0.9995

$IE_{M\left(T^{\lambda^{d}}\right)}$	P	$x_{0}$	$y_{0}$	$z_{0}$	$R^{2}$
$IE_{M\left(M_{2}^{\lambda^{d}}\right)}$	$E^{2b}$	−358.21311	5.90356	1.0191	0.9996
$IE_{M\left(M_{2}^{\lambda^{d}}\right)}$	$E^{es}$	−5.61257	0.51405	0.53482	0.9965
$IE_{M\left(M_{2}^{\lambda^{d}}\right)}$	$E^{te}$	−440.91722	6.934	1.02202	0.9996

The exponential model is chosen for it accurately captures the rapid, non-linear growth of entropy as molecular interaction energies increase in large zeolite frameworks. Unlike polynomial models, the exponential model reflects the exponential increase in the complexity and entropy, providing a more accurate and meaningful representation of their relationship, particularly in complex systems where small energy changes lead to significant increases in entropy.

**FIGURE 4:**
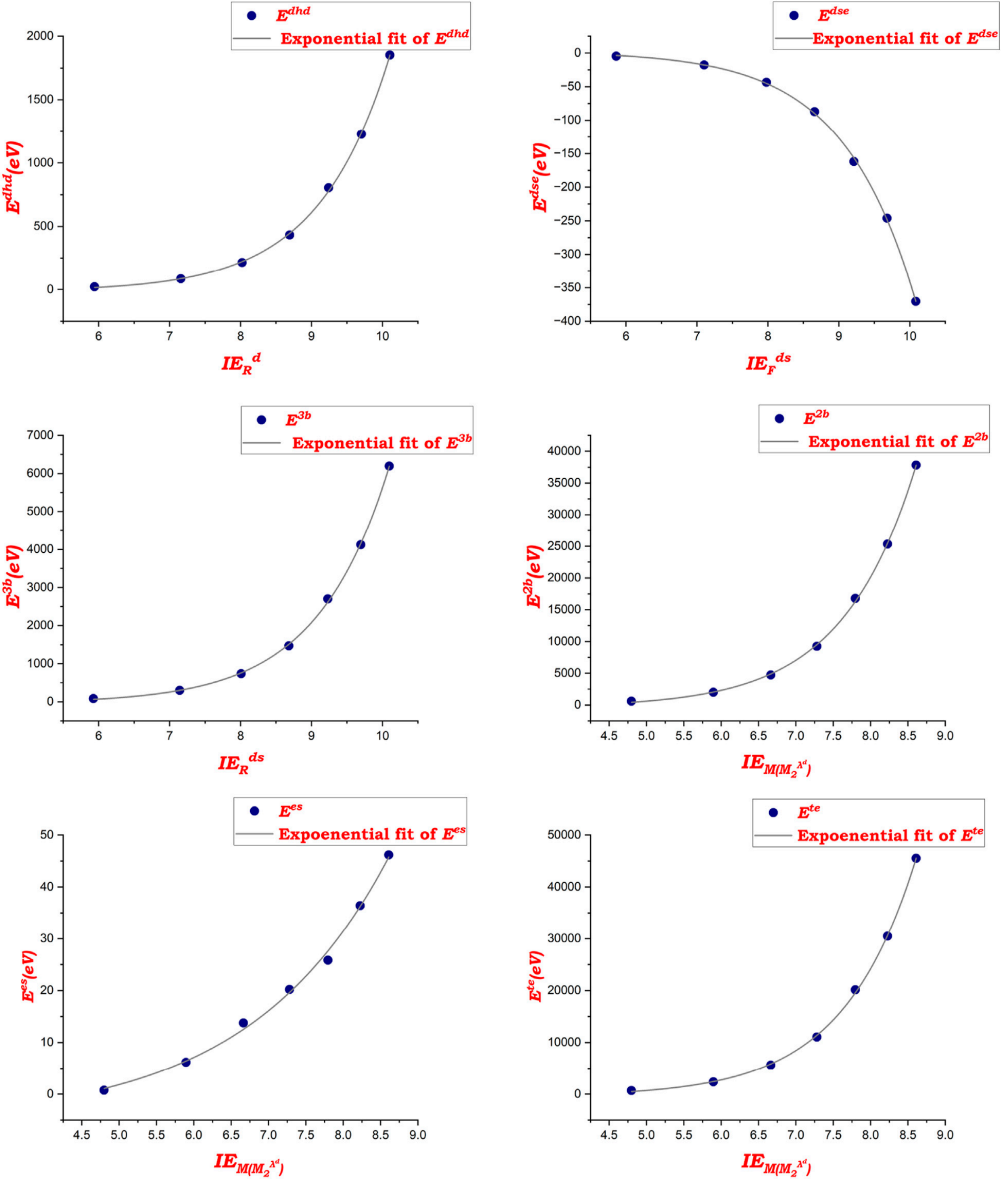
Exponential fitting of bond and spectral information entropy with properties of AWW zeolite.

Table 4 presents the significant coefficients for exponential fit of entropies with molecular energies. The proposed model obtained from Table 4 shows that $IE_{M\left(M_{2}^{d}\right)}$ could be utilized for forecasting molecular properties such as $E^{2b}$ , $E^{es}$ , and $E^{te}$ . The relationship between information entropy and complexity of zeolite AWW demonstrates that increased entropy results in more intricate and flexible zeolite AWW structure. Figure 4 depicts the exponential fit of the measured bond information, spectral entropies, and molecular energies for the zeolite AWW. The topological indices and information entropies were calculated using MATLAB software, and the statistical OriginLab package was used for evaluating the correlations between experimental data and various information entropies.

Table 5 presents a comparison between properties and predicted molecular interaction energies for AWW zeolites. The table also highlights consistent trends across different zeolite frameworks, particularly for larger structures, demonstrating the robustness of entropy-based models in capturing complex molecular interactions and effectively predicting energy properties.

**TABLE 5:** Comparison of molecular interaction energy values for AWW zeolite using the force-field method and predictive model AWW(j,k,l) for j=k=l .

Zeolites		(2,2,2)	(3,3,3)	(4,4,4)	(5,5,5)	(6,6,6)	(7,7,7)	(8,8,8)
$E^{\mathrm{dhd}}$	Force field	22.57785	85.61961	214.2766	431.884	804.6665	1227.29	1851.758
	Predicted	15.7257	86.1592	222.3082	445.5162	848.8865	1237.7453	1848.8865
$E^{\mathrm{dse}}$	Force field	−4.79332	−17.6518	−43.616	−87.2846	−161.772	−246.129	−370.502
	Predicted	−3.5503	−17.7207	−45.148	−90.017	−156.4551	−248.52	−370.23
$E^{3b}$	Force field	83.2212	301.2218	738.1260	1469.9167	2698.1572	4122.0882	6194.4345
	Predicted	64.1855	302.645	761.412	1510.6639	2619.7381	4157.2141	6191.4708
$E^{2b}$	Force field	592.2638	1996.6792	4730.2483	9235.6949	16768.2665	25333.1150	37810.5359
	Predicted	426.8851	2040.8758	4894.134	9500.7099	16305.1658	25468.9412	37831.1494
$E^{es}$	Force field	0.7434	6.1657	13.7597	20.2031	25.8579	36.4116	46.1900
	Predicted	1.0797	6.4146	12.5324	19.6382	27.6452	36.2446	45.7817
$E^{te}$	Force field	694.0131	2372.035	5652.795	11070.41	20135.18	30472.78	45532.42
	Predicted	494.2282	2425.8439	5849.416	11387.6812	19581.66	30631.7666	45556.2758

### 4.3 Global chemical reactivity descriptors and spectral properties of AWW zeolite

Assessing global chemical reactivity descriptors such as ionization potential (IP), electron affinity (EA), hardness (η) , chemical potential (μ) , softness (σ) , and electrophilicity index (ω) is challenging for complex materials like zeolites due to the computational and experimental requirements, high resource demands, and the impracticality of DFT and quantum mechanical calculations. Studies use global chemical reactivity descriptors to evaluate the electron-attracting ability, negative of electronegativity, reflects the resistance to electron density changes, and molecule’s tendency (Hemelsoet et al., 2007; Yong et al., 2014; Manda et al., 2024; Gázquez and Sen, 1993; Pearson, 1988; Padmanabhan et al., 2007). The HOMO (highest occupied molecular orbital) and LUMO (lowest unoccupied molecular orbital) energies are closely related to the ionization potential (IP) and electron affinity (EA) in quantum chemistry (Yin et al., 2021). Therefore, using the HOMO and LUMO energies, several important chemical quantum descriptors can be calculated using the given below equations:

$$IP=-E_{\text{HOMO}},$$ (13)

$$EA=-E_{\text{LUMO}},$$ (14)

$$\eta =\frac{IP-EA}{2},$$ (15)

$$\mu =-\left(\frac{IP+EA}{2}\right),$$ (16)

$$\sigma =\frac{2}{\eta }.$$ (17)

$$\omega =\frac{\mu^{2}}{2\eta }.$$ (18)

In this section, we propose the measurement of the global chemical reactivity descriptors using the eigen values of the zeolite graph as the HOMO–LUMO gap. Therefore, we employ the graph-theoretic approach to compute the HOMO–LUMO gap, which involves modeling the zeolite structure as a graph, constructing adjacency, and calculating the eigenvalues of the zeolite matrix. The difference between the maximum negative and minimum positive eigenvalues of the characteristic polynomial gives the HOMO–LUMO gap, which is a key indicator of the molecule’s electronic properties and reactivity (Arockiaraj et al., 2022a; Fowler and Pisański, 2010; Dias and Guirgis, 2002; Aihara, 1999; Bacalis and Zdetsis, 2009; Mushtaq et al., 2022; Ghosh, 2019).

The HOMO–LUMO gap in the zeolite structure reflects their chemical reactivity. A larger HOMO–LUMO gap typically indicates greater stability and lower chemical reactivity as it requires more energy to excite an electron from the HOMO to the LUMO. Conversely, a smaller gap implies higher chemical reactivity as less energy is needed for electronic transitions, facilitating interactions with adsorbates or reactants (Yin et al., 2021). Furthermore, the spectral properties (SP) of chemical structure such as spectral diameter (SD), graph-energy (GE), and spectral entropy (SGE) (Arockiaraj et al., 2022a; Balasubramanian, 2023b; Balasubramanian, 2023c) are computed using the eigen values of characteristic polynomials for the different AWW zeolites listed in Table 6. The global chemical reactivity descriptors are calculated using Equations 13–18, and the summarized values in Table 6 are measured using Python code. One can observe from Table 6 that as the AWW zeolite structure increases, the global descriptors such as hardness, chemical potential, and electrophilicity index decrease. On the other hand, the softness of the AWW zeolite structure is increasing.

**TABLE 6:** Spectral properties and global reactivity descriptors of zeolite AWW(j,k,l) .

Zeolites	(2,2,1)	(2,3,1)	(2,2,2)	(3,4,1)	(3,6,1)	(3,3,3)	(4,5,1)	(4,4,4)
$\Delta_{HL}/\beta$	0.1666	0.1112	0.0246	0.0448	0.0117	0.003	0.0004	0.0059
SD	6.995	7.0574	7.4448	7.1454	11.4739	7.717	7.181	7.8028
GE	214.4309	295.5472	349.7192	594.3956	1166.9287	1144.112	993.6112	2,303.201
SGE	2.000962	2.191812	2.252636	2.489498	2.656461511	2.753511	2.709569	3.064081
η	0.0833	0.0556	0.0123	0.0224	0.00585	0.0015	0.0002	0.00295
μ	0.0833	0.0556	0.0123	0.0224	0.00585	0.0015	0.0002	0.00295
σ	24.0096	35.97122302	162.6016	89.28571	341.8803	1333.333	10,000	677.9661
μ	0.000289	8.59398 $\times 10^{-5}$	9.3 $\times 10^{-7}$	5.62 $\times 10^{-6}$	1 $\times 10^{-7}$	1.69 $\times 10^{-9}$	4 $\times 10^{-12}$	1.28 $\times 10^{-8}$

Here $\Delta_{HL}$ is the difference of minimum positive and maximum negative eigenvalues with respect to zeolite adjacency matrix.

β is the Hückel energy parameter.

Table 7 presents molecular energy properties of various AWW zeolite structures, serving as a benchmark to validate predictive models developed using the measured spectral values of AWW zeolite. It establishes a critical link between computed molecular energies and spectral values, demonstrating the reliability of theoretical calculations in predicting energy properties. The table also highlights how structural variations in zeolites influence energy behavior, emphasizing the practical utility of spectral and entropy-based analyses in evaluating the stability and reactivity.

**TABLE 7:** Molecular interaction energies of various AWW zeolites.

Zeolites	$E^{2b}$	$E^{3b}$	$E^{\mathrm{dhd}}$	$E^{es}$	$E^{\mathrm{dse}}$	$E^{te}$
AWW(2,2,1)	322.066035	42.623374	10.428624	0.066132	−2.305049	372.879116
AWW(2,3,1)	489.336864	66.449394	16.444560	0.088597	−3.592838	568.726576
AWW(2,2,2)	592.263894	83.221202	22.577852	0.743495	−4.793315	694.013128
AWW(3,4,1)	1839.169786	274.806936	75.990215	3.427539	−15.775537	2177.618938
AWW(3,6,1)	2777.468110	419.753403	116.470960	5.347468	−24.082915	3294.957026
AWW(3,3,3)	1996.679237	301.221814	85.619606	6.165783	−17.651809	2372.034631
AWW(4,5,1)	3098.551299	471.421336	131.088470	6.261098	−27.040602	3680.281600
AWW(4,4,4)	4730.248377	738.126087	214.276638	13.759779	−43.616033	5652.794848

The data in Table 8 summarize the significant coefficients of the developed polynomial fit Equation 19. By using the molecular energies and spectral values of zeolite AWW listed in Tables 6, 7, the models demonstrate their effectiveness in predicting potential energies, as evidenced by the high $R^{2}$ values, which indicate strong relationships.

$$P=a+b\left(SP\right)+c{\left(SP\right)}^{2}.$$ (19)

where P , a , b, and c represent the property and coefficients of the models, and SP denotes the spectral properties of the AWW zeolite structures.

**TABLE 8:** Significant coefficients for polynomial fit of spectral properties with molecular energies.

P	SP	a	b	c	$R^{2}$
$E^{2b}$	SGE	4719.62372	−6503.2536	2117.1465	0.92393
$E^{3b}$	GE	−54.3851	0.48815	6.4209 $\times 10^{-5}$	0.926
$E^{es}$	SGE	47.01358	−47.2824	11.87595	0.99352
$E^{\mathrm{dhd}}$	GE	−16.3696	0.13496	1.55871 $\times 10^{-5}$	0.93377
$E^{\mathrm{dse}}$	GE	3.21832	−0.02788	3.37231 $\times 10^{-6}$	0.93221
$E^{te}$	SGE	5968.9825	−8055.11231	2587.39569	0.92488

**FIGURE 5:**
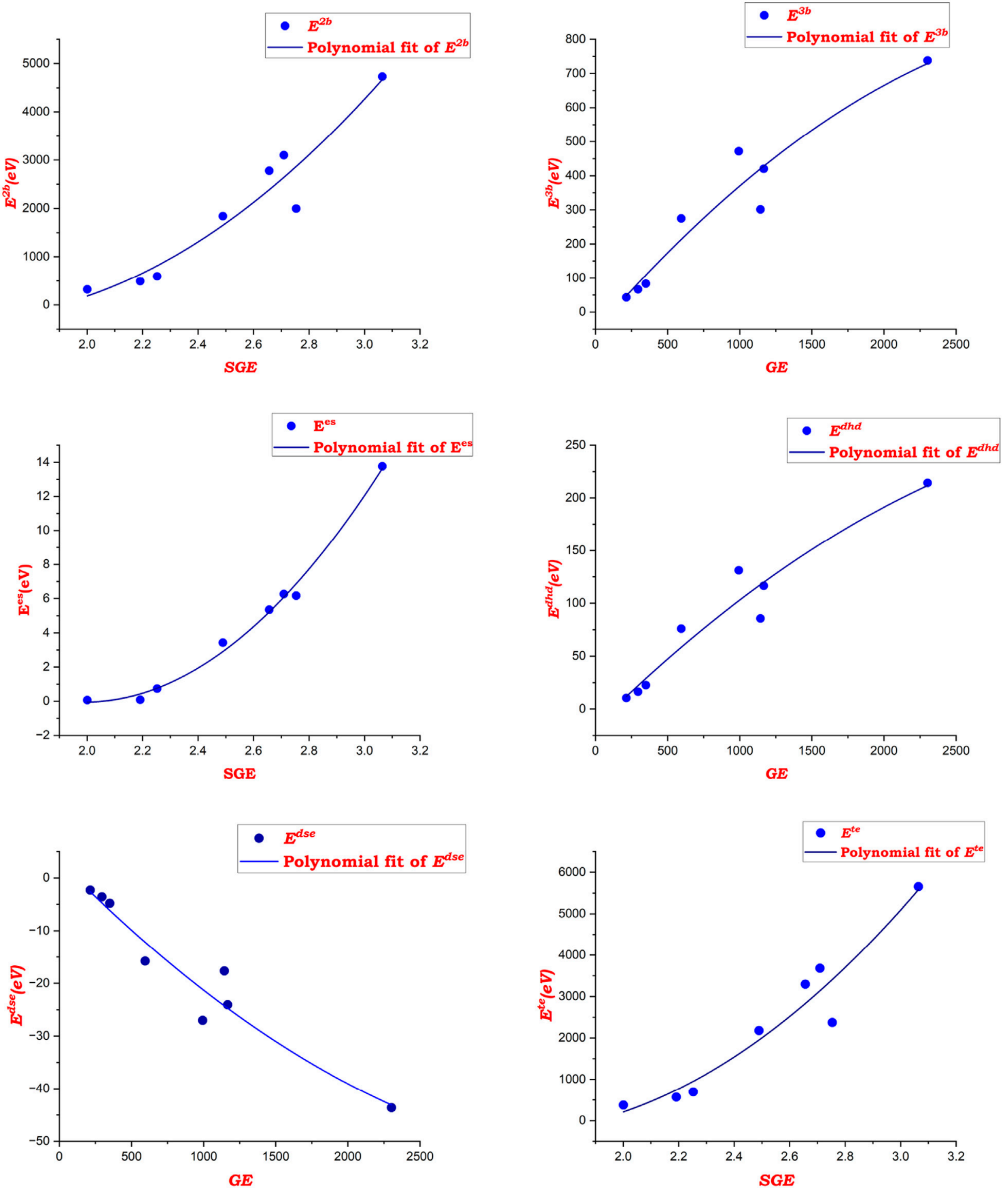
Polynomial fitting of spectral values with properties of AWW zeolites.

Figure 5 highlights the polynomial fitting of spectral values with AWW zeolite properties, demonstrating a strong correlation between spectral entropy and molecular characteristics. The model shows high accuracy, validating the use of spectral properties for predicting molecular energies.

The obtained data, such as the HOMO–LUMO gap and spectral information entropy, directly relate to the applications of AWW zeolites. A smaller HOMO–LUMO gap suggests higher reactivity, enhancing catalytic activity, while spectral information entropy reflects the structural complexity, influencing adsorption capacity through pore connectivity and active site diversity. These comparisons contextualize the findings, linking structural properties to their functional performance in industrial applications.

## 5 Conclusion

Predicting the characteristics of complicated multicomponent zeolite materials and extremely high-molecular weight systems of molecules is an essential step in the development of QSPR advances in technology. We have developed the degree-sum-based molecular descriptors for zeolite AWW, analyzing three information entropies obtained from topological indices and their spectral aspects. Furthermore, we proposed the exponential regression and polynomial models to predict its potential energies using the computed information measures. The eigenvalue difference approximation of the HOMO–LUMO gap reduces computational demands compared to more complex DFT calculations, enabling faster reactivity predictions in large systems or high-throughput screening. The results of the present investigation illustrate the usefulness and efficacy of the quantitative structure–property relationship approach for the prediction of an extensive variety of properties of the zeolite material.

## Data Availability

The raw data supporting the conclusions of this article will be made available by the authors, without undue reservation.
